# The Potential Effects of Red Wine and Its Components on Neurocognitive Disorders: A Narrative Review

**DOI:** 10.3390/nu16203431

**Published:** 2024-10-10

**Authors:** Virginia Boccardi, Luca Tagliafico, Angelica Persia, Elena Page, Silvia Ottaviani, Anna Laura Cremonini, Consuelo Borgarelli, Livia Pisciotta, Patrizia Mecocci, Alessio Nencioni, Fiammetta Monacelli

**Affiliations:** 1 Division of Gerontology and Geriatrics, Department of Medicine and Surgery, University of Perugia, 06123 Perugia, Italy; 2 Department of Internal Medicine and Medical Specialties, University of Genoa, 16132 Genoa, Italy; 3 IRCCS Ospedale Policlinico San Martino, 16132 Genoa, Italy; 4 Division of Clinical Geriatrics, Department of Neurobiology, Care Sciences and Society, Karolinska Institutet, 171 77 Stockholm, Sweden

**Keywords:** clinical studies, dementia, neurocognitive disorders, neurodegenerative diseases, polyphenols, preclinical studies, prevention, red wine, resveratrol

## Abstract

Background: The aging population is associated with a net increase in the incidence and prevalence of chronic-degenerative diseases, particularly neurocognitive disorders. Therefore, the identification of preventative strategies to restrain the burden of such chronic conditions is of key relevance. Red wine and its components have accumulated evidence regarding their positive effects in terms of neurological pathologies associated with neurocognitive symptoms. Methods: Based on this background, the present narrative review aims to summarize the state-of-the-art evidence on the effects of red wine and its components on neurocognitive disorders in both preclinical and clinical settings. Results: The main findings highlight a protective effect of wine polyphenols present in red wine on dementia in different preclinical models of cognitive decline. The current translational clinical evidence remains uncertain, especially considering the risk-to-benefit ratio of alcohol consumption on brain health. Conclusions: Given the overall health risks associated with red wine consumption and consistent with the prevailing guidelines in the literature, there is insufficient evidence to support light-to-moderate red wine consumption as an effective strategy for preventing these diseases. However, the largely preclinical findings on polyphenols derived from red wine remain of significant interest in this context.

## 1. Introduction

The increase in lifespan over the last few decades has been characterized by the increase in age-related diseases in the global population, such as cognitive decline and dementia [1]. Currently, more than 55 million people worldwide have dementia, with the disease being the seventh leading cause of death and one of the major causes of disability and dependency among older people [2]. The etiology of age-related cognitive decline is complex and multifactorial [3]. Brain volume and weight generally begin to decline around age 40, and the process is further accelerated after age 70, with potential consequences for cognitive functions [4,5]. Neuroinflammation is a key player in brain aging and is mediated by inflammatory signaling to the central nervous system as an indirect consequence of systemic inflammation [6]. The term “inflammaging” is commonly used to indicate the systemic low-grade inflammation present during aging [4], sustained by cellular-senescence-associated inflammation, changes in the gut microbiome, endocellular mechanisms’ dysregulation, and external stressors [7]. Chronic stresses, but also single experiences of acute stress, may have long-term consequences for brain physiopathology. Recent studies have shown that acute or subacute stress can induce rapid and sustained alterations in neuroarchitecture, synaptic function, and behavior, leading to neurodegeneration [8].

Dementia is a broad term used to describe a range of major neurocognitive disorders associated with a decline in memory, thinking, and social abilities that are severe enough to interfere with daily life. The most common forms of dementia are Alzheimer’s disease (AD), vascular dementia (VaD), Lewy body dementia (LBD), Parkinson’s disease dementia (PDD), and frontotemporal dementia (FTD) [2].

Although each neurodegenerative disease has its own clinical and neuropathological characteristics, they all share the pathological accumulation and aggregation of disease-specific proteins that lead to neuron and synapse loss in specific brain areas [1].

Nowadays, treatments for persons with cognitive decline for most of the neurodegenerative-associated diseases can only try to manage cognitive and behavioral symptoms, failing to affect the underlying pathology [1]. This lack of disease-modifying treatments further emphasizes the importance of finding preventive strategies able to slow down neurodegenerative processes [9].

In this context, dietary habits have been proposed as a potential alternative to prevent age-related cognitive decline [10]. As shown in the literature, compliance with a Mediterranean diet has been associated with slower cognitive decline and a reduction in the incidence of mild cognitive impairment (MCI) and dementia [11,12]. This effect of neuroprotection is mediated by polyphenol-enriched foods in the Mediterranean diet, which seem to exert an additive or synergistic effect on brain health when consumed in a plant-centric diet [13,14,15].

The beneficial effects of a polyphenol-enriched diet against oxidative stress and chronic inflammation in age-related diseases have been widely investigated in the literature [1]. Polyphenols exert their antioxidant effect by directly scavenging radicals in lipid peroxidation and by interacting with various signaling targets implicated in most risk factors for inflammation and neurodegeneration [3,9,16].

The interaction between phenolic compounds and endothelial nitric oxide (NO) has a key role in the improvement of cerebrovascular function, as well as in other mechanisms, such as the interaction with the gut microbiota and glucoregulation (note that impaired glucose tolerance is associated with poorer cognition) [3,17,18]. A polyphenol-enriched diet has demonstrated the improvement of cognitive function and brain health by enhancing the circulation of pro-cognitive neurotrophic factors [3]. Brain-derived neurotrophic factor (BDNF) and nerve growth factor (NGF) are neurotrophins well known to promote neuronal growth and survival and modulate synaptic plasticity [19]. There are no clear conclusions regarding the impact of polyphenol intervention on biomarkers, such as BDNF, NGF, amyloid beta (Aβ), and tau proteins [19]. However, some studies have shown an overall improvement in cognitive performance in young and/or middle-aged subjects following polyphenol-rich supplementation [20]. The neuroprotective effect of phenolic compounds seems to be more promising in a younger population, while in older adults, the results remain controversial [20]. To be able to cross the blood–brain barrier (BBB) and to exert a significant effect on cognitive health, polyphenol bioavailability rates need to be between 9% and 43% [20]. In this scenario, studies enrolling older adults have encountered more difficulties in demonstrating the beneficial effect of polyphenols, as the bioavailability rate is strongly dependent on the ingested dose and on gut microbiota (altered in older adults) [3].

The study “Invecchiare inCHIANTI” investigated the association between total urinary polyphenols (TUPs), total dietary polyphenols (TDPs), and cognitive decline in older adults without dementia [21]. In this population-based study, high concentrations of TUPs were associated with an approximately 47% lower risk of cognitive decline in global cognitive function and an approximately 48% lower risk of cognitive decline in attention [21]. High concentrations of TUPs were not associated with a lower risk of cognitive decline in executive function. No significant association was found between TDPs and any cognitive test [21].

Grapes (Vitis vinifera) are one of the richest sources of polyphenols, containing a unique combination of bioactive dietary phenols with pleiotropic biological effects [8,22]. While red grape polyphenols possess an important antioxidant capacity in vivo, the presence of the alimentary matrix (grape juice) may interfere with this property, as more than 70% of grape phenolics can be retained in skins and seeds [22,23].

In their systematic review, Thaung Zaw and colleagues emphasized the role of resveratrol as a promising candidate for cognitive enhancement in older adults, with cognitive benefits on working and episodic memory seen at doses ranging from 150 to 200 mg/day [24,25]. However, another two meta-analyses did not find significant cognitive benefits from resveratrol supplementation in the general population or only reported a slight improvement in delayed recognition memory with a modest effect size [24,26,27].

The neuroprotective effect of red wine resides in its richness of specific grape phenolic compounds (e.g., quercetin, myricetin, catechins, tannins, anthocyanidins, resveratrol, and ferulic acid), whose intake is associated with a lower incidence of cognitive decline [28,29,30].

Alcohol is known to be an enzyme inductor. However, enhanced antioxidant enzyme activities after wine consumption seem to be due to the polyphenols in red wine, not the alcohol [31]. The cognitive impairment caused by ethanol exposure may be significantly reduced by quercetin, as its chronic administration seems to have neuroprotective effects in animal models. In Amanpreet Singh and colleagues’ study, quercetin was shown to protect brain cells against oxidative stress by reducing lipid peroxidation and increasing glutathione, superoxide dismutase (SOD), and catalase levels in aged mice [32]. Furthermore, flavanols in red wine can enhance hippocampal vascular plasticity through their positive action on blood pressure control, cerebral blood flow, endothelial function, and reduction of LDL cholesterol oxidation [33,34,35].

Another class of phenolic compounds present in red wine are stilbenes, whose pro-cognitive effects are mediated by their estrogenic activity on estrogen receptors (alpha and beta) expressed in brain areas important for memory and higher-order cognitive function, such as the hippocampus and prefrontal cortex [10].

However, the relationship between red wine consumption and cognitive function remains complex, controversial, and uncertain. It has been suggested that there is a U-shaped relationship between alcohol consumption and cognitive function. Low-to-moderate red wine intake is associated with better global cognition scores and a reduced risk of developing dementia, specifically AD, while excessive and chronic alcohol consumption are well-established risk factors for early-onset dementia and multiple chronic diseases [36,37,38].

Considering the above literature, this review aims to summarize the effects of red wine and its components on neurocognitive disorders, evaluating state-of-the-art findings from both preclinical and clinical studies.

## 2. Methods

This narrative review was based on a search of the MEDLINE database for articles in English published from 1 January 2000 to 31 December 2023, regarding the effects of red wine and its components on neurocognitive disorders.

The search terms for the literature review included the following: red wine, polyphenol, dementia, cognitive impairment, cognitive decline, neurodegenerative, AD, and neurocognitive disorders, in all possible combinations. Both preclinical and clinical studies were evaluated. We excluded articles that did not relate to these keywords, as well as narrative and editorial reviews, studies on synthetic compounds derived from red wine, and their technological delivery methods, such as nanodots.

The initial phase of article selection was conducted by the first four authors, who unanimously agreed on which articles to include and exclude. In the subsequent phase, the first three authors each focused on a specific area: wine characteristics, preclinical data, and clinical data. A comprehensive evaluation of the findings was then conducted by all the remaining contributors to the review, incorporating the articles selected.

**Figure 1:**
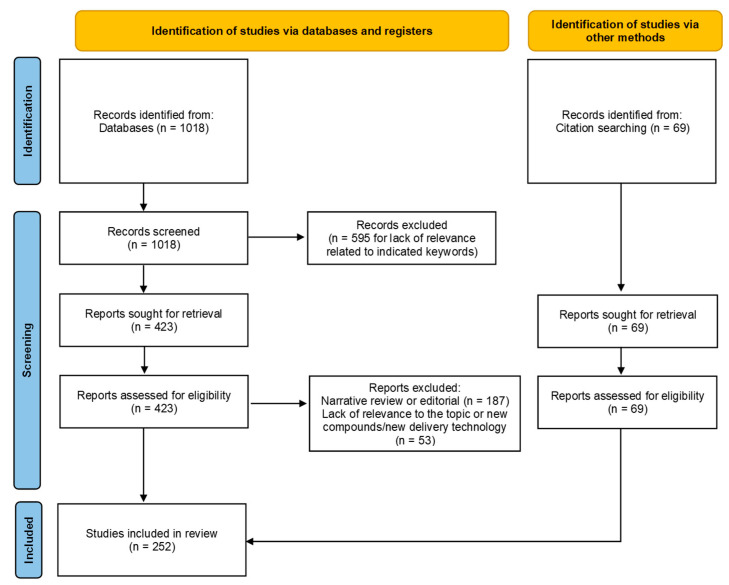
PRISMA flow diagram for the literature search.

Figure 1 illustrates the selection process with a PRISMA flow diagram [39].

We included 183 suitable studies from 1018 articles initially identified in the MEDLINE database, as well as 69 articles that were not present in the reference database. Specifically, the articles excluded during the screening stage were omitted due to a lack of relevance to the topics covered by the selected keywords. Regarding articles excluded for not meeting eligibility criteria, the primary reasons were as follows: 187 were narrative reviews or editorials, while the remainder were excluded for not aligning with the review’s topics of interest, involving synthetic compounds or new technological delivery systems, as summarized by the selected keywords.

The paper not identified in the initial literature evaluation was found in the bibliography of a selected article. Additionally, some articles not retrieved through MEDLINE were included, particularly in the section on the composition and chemical characteristics of red wine.

In addition to our main objective of describing the effects of red wine and its components on neurocognitive disorders in both preclinical and clinical settings, we aimed to begin the text with a paragraph outlining the general characteristics of red wine. This was intended to provide clearer context for the subsequent discussion of current literature related to the primary objective. To achieve this, we utilized both the literature identified in the main search and studies found through citation searching, as noted in the flow diagram under identification of studies via other methods.

To enhance the rigor of the search and writing process, we used the Scale for the Assessment of Narrative Review Articles (SANRA) as a methodological guideline [40]. SANRA scores measure the quality of a narrative review based on justification of the article’s importance (item 1), a statement of aims or questions (item 2), a description of the literature search (item 3), referencing (item 4), scientific reasoning (item 5), and appropriate presentation of data (item 6). Although SANRA is designed for use by editors and peer reviewers, it can be applied during the drafting phase of an article, as in our case.

## 3. Results

### 3.1. Red Wine General Characteristics

Red wine is an alcoholic beverage obtained through the fermentation process of crushed grapes [41]. The grape variety and the different vinification methods, climate, country, and year influence the color and characteristics of the final product, making it unique [41,42]. Beyond being mostly composed of water (about 86%) and ethyl alcohol (9–15%), wine also contains a wide range of other molecules [43]. These include monosaccharides, such as glucose and fructose, as well as varying levels of micronutrients, such as potassium, calcium, iron, magnesium, and copper. It is also rich in certain B-group vitamins, organic acids, and polyphenols, which makes red wine particularly interesting from a nutritional standpoint [41,43].

Polyphenols are a large family of compounds of plant origin involved in the response of plants to various types of biotic and abiotic stresses [44]. With their aromatic rings and hydroxyl groups, phenols are a class of compounds that are known to be antioxidants [45]. Phenols can be complex, heavy, condensed tannins or simple, tiny molecules with a single aromatic ring [46].

In red wine, more than 100 polyphenols have been identified [41], mainly contained in the grains (in the skins and seeds) and in the stalks. The presence of polyphenols in grapes is influenced by the ripening conditions of the bunch, as well as by exposure to the sun, the geographical position, and the type of soil; in addition, winemaking conditions, such as maceration and fermentation, are very important, affecting the extraction of the various constituents of wine and their reactions [47]. The phenolic compounds have a significant impact on the quality of wine and grape juice. These substances affect the wine and grapes’ color, as well as their astringency [48].

The strain or strains of yeast used in winemaking during the fermentation affect the aroma, stability, and color of wines, as they can influence the quality and quantity of phenols present in the wine in several ways: directly, through adsorption on the cell wall or enzymatic activity, and indirectly, through the production of primary and secondary metabolites and fermentation by-products [49].

In general, the maceration processes underlying the vinification of red wine facilitate the extraction and diffusion of polyphenols into the juice, resulting in red wine having polyphenol concentrations up to 10 times higher than those found in white wines [50]. The quantity of polyphenols in wine, although varying considerably, is estimated to be around 900–2500 mg/L in red wines and 190–290 mg/L in white wines. The variability in polyphenolic composition appears to be significant in determining its effects [42].

Chemical structure, biological function, and origin have all been used to categorize polyphenols [51]. More commonly, polyphenols are grouped into two broad categories: flavonoids (e.g., flavones, flavan-3-ols, flavanols, anthocyanins, tannins, condensed tannins, and hydrolysable tannins) and non-flavonoids (e.g., hydroxybenzoic acids, hydroxycinnamic acids, and resveratrol) [52]. In the last 30 years, scientific research has identified the main polyphenols contained in grapes and wine thanks to the use of analytical techniques based on mass spectrometry (MS) and, in particular, high-performance liquid chromatography coupled with mass spectrometry (HPLC-MS), which have made possible the identification of even highly polymerized polyphenols [53].

Flavones are a subgroup of flavonoids, which exhibit a distinctive structure characterized by three functional groups: hydroxy groups, carbonyl groups, and conjugated double bonds between C2 and C3 in the flavonoid skeleton. These compounds were found in grape skin and wine in both aglycone and glycoside forms. One specific flavone mentioned in the context of grapes is luteolin. Luteolin is found in grape skin, and its levels can vary within the range of 0.2 to 1 mg/L in grapes [52].

Red wine contains high levels of flavanols, particularly catechins and proanthocyanidin dimers [54]. In wine, flavanols (catechins) can reach concentrations of up to 300 mg/L [50]. However, the content of some flavanols is often underestimated because, generally, the methods used (e.g., HPLC) evaluate only monomers, dimers, and trimers of proanthocyanidins [54].

Red wine contains 45 mg of flavonols/L, with a described maximum content of 60 mg/L. These compounds are primarily found in the skin and leaves of the grape plant, as their synthesis is stimulated by light [50,55]. They are present in glycosidic form, which is bound to a sugar (such as glucose or rhamnose), but they can also contain other sugars, such as galactose, arabinose, xylose, or glucuronic acid. Flavonols are present in both white and red wines, and they play a key role in influencing the perception of bitterness and astringency [55]. In addition to providing protection against ultraviolet radiation, they contribute to the co-pigmentation function, together with anthocyanins [56]. Fisetin and quercetin are two key flavonols known for their potential pharmacological relevance [57]. Morin and rutin are flavonols that are considered important bioflavonoids [58,59].

At the beginning of the 20th century, water-soluble pigments, called anthocyanins, were recognized in red grape berries [54,60]. Anthocyanins are mostly found in the skin and are responsible for the red, purple, and blue coloration of flowers and fruit [54]. In wine, they contribute to key organoleptic properties, such as astringency and aroma [61]. Anthocyanins are classified according to the number and position of the OH groups on the flavonoid molecule. To date, more than 600 anthocyanin compounds have been identified [54]. In young, full-bodied red wines, free anthocyanin concentrations typically range from 500 mg/L to 2000 mg/L, with rosé wines typically having concentrations between 20 and 50 mg/L [62].

Another significant subgroup of phenols found in red wine is tannins, which also contribute to astringency and are involved in reactions that cause browning. The two primary groups into which they can be divided are hydrolyzable and condensed tannins [52].

Condensed tannins (or proanthocyanidins) are produced as the product of the flavonoid biosynthetic pathway and are oligomers or polymers of monomeric flavan-3-ols. Catechin and epicatechin are the constituent elements of proanthocyanidins, and the degree of polymerization of proanthocyanidins can range from 3 to 11. The elementary units of these polymeric flavan-3-ols are connected by C-C bonds, and occasionally C-O-C bonds. Proanthocyanidins from grape seeds are used as dietary supplements and confer astringency, bitterness, acidity, sweetness, salivary viscosity, aroma, and color formation [63].

Hydrolyzable tannins are a type of tannin with a structure consisting of basic units represented by gallic and ellagic acids, typically esterified with glucose or related sugars. It is interesting to note that hydrolyzable tannins are not naturally found in grapes. Instead, they are extracted from wooden barrels during the aging process of wine. As a result, the presence of hydrolyzable tannins in wine is considered a marker of maturity for certain types of wines. The final content of hydrolyzable tannins in wine may range from 0.4 to 50 mg/L, reflecting the diverse conditions under which wines are produced and aged [52].

Non-flavonoid polyphenols are mainly phenolic acids, which are divided into two large families: hydroxybenzoic acids (the most important is gallic acid, which plays a role in the aging and color change phases of wine) and hydroxycinnamic acids (in free form or in the form of esters).

Chlorogenic acid and caffeic acid are the main polyphenolic representatives of hydroxycinnamic acids [64]. P-coumaric acid, also named trans-4-hydroxycinnamic acid, is a phenolic compound, which we will discuss more later [65].

A third group of non-flavonoids is that of stilbenes, where we find piceid and resveratrol [66]. Resveratrol is mainly contained in the skin of ripe red grapes and has been extensively studied due to its potential beneficial effects against aging and chronic degenerative diseases [47,67].

Resveratrol, known since ancient times, gained widespread attention in the early 1990s following the publication of the “French Paradox,” an epidemiological study [68]. Resveratrol (3,5,4′-trihydroxystilbene) is a potent polyphenol antioxidant widely distributed in over 70 species, including blackberries and peanuts. However, its primary sources are grapes and their derivatives [69,70]. It is known to be especially concentrated in grape skin. The content of this phytoalexin in grapes and grape-derived products, including wine, varies from region to region and from year to year [68,71]. The average concentration of total resveratrol in red wine is 7 mg/L, in rosé wine, it is 2 mg/L, and in white wine, it is 0.5 mg/L [54], with the levels influenced significantly by vine cultivation methods, climatic and geographical factors, and the wine production processes [72]. Perhaps the most researched secondary metabolite in plants is resveratrol [68], which, similar to other stilbenes, is produced by plants in reaction to stressors, such as infections and UV radiation [71]. Resveratrol is gaining a lot of interest right now because of its potential for medicinal use. Its use has been connected to several health benefits, such as anti-aging, neuroprotective, anti-inflammatory, and antioxidant properties, as we will also discuss further in the context of neurocognitive disorders [69,73]. Despite having significant beneficial biological effects on human health, resveratrol has a poor pharmacokinetic profile because of its low water solubility, poor chemical stability during digestion, and low bioavailability. Resveratrol’s sensitivity to sulfation and glucuronidation during phase II reactions in the gut and liver, as well as its significant metabolism by intestinal bacteria, appear to be the causes of its decreased oral bioavailability [46,74].

Studying polyphenols, we must consider that the quantitative parameter is not the only significant one. The qualitative composition of wines is also an important factor in evaluating their positive or negative effects. Some polyphenols do not act synergistically but rather additively, and in some cases, they have an opposite effect. Imbalance among polyphenolic species can increase or reduce their beneficial effects. The presence of (+)-catechin reduces the synergy between resveratrol and quercetin. This could explain the differing results obtained from various studies: some studies have shown that moderate consumption of red wine in humans or animal models has reduced the risk of colon cancer, while others have shown no effect. Therefore, the phenolic composition cannot be underestimated when examining the effects of wine. When studied separately, some of these polyphenols that are present in significant quantities exhibit strong activity. This is particularly true for the well-known chemopreventive compounds resveratrol, quercetin, (+)-catechin, and gallic acid [42,75,76].

The relationship between dietary polyphenol consumption and its biological effects remains largely unclear due to the many factors involved when polyphenol-rich foods are consumed. One of the most significant variables is the chemical structure of polyphenols, which has a major impact on their absorption and bioavailability [50]. In fact, most of the polyphenols found in foods are represented by glycosylated compounds, esters, or polymers (except for proanthocyanidins—see below). Once ingested, they cannot be absorbed in their native form. Instead, they undergo structural modifications by enzymes of the small intestine and colon microbiota, leading to the production of metabolites [77].

These modified molecules are absorbed and then further modified in the liver through methylation, glucuronidation, and sulfation reactions and, once conjugated, can bind to albumin in the bloodstream. The elimination of these metabolites is usually very rapid, so daily (even repeated) consumption of foods rich in polyphenols is necessary to maintain detectable and potentially effective serum concentrations of these compounds.

Proanthocyanidins differ from other polyphenols due to their highly polymerized structure and their high molecular weight, which makes them resistant to the gastric acid environment and digestion in the small intestine [78,79]. It has been hypothesized that proanthocyanidins perform their action locally at the level of the intestinal barrier, which is largely subjected to oxidative stress and other toxic insults [80]. The bioavailability of polyphenols and their consequent biological activity are, therefore, highly dependent on the processes of intestinal digestion, absorption, hepatic conjugation and elimination, and metabolism by the microflora, which can vary greatly in terms of efficiency from individual to individual [50]. For instance, just 1% to 2% of anthocyanins ingested with food are able to maintain their original molecular structure [46,54].

The bioavailability of dietary polyphenols is affected by the chemical and physical characteristics of the natural matrix in which they are found. Additionally, it is influenced by the digestive process and the metabolism of intestinal enzymes. Furthermore, the intestinal microbiota plays a role in altering the bioactivity and bioavailability of polyphenols [81].

See Table 1 for the polyphenol content of the red wine.

### 3.2. General Neuroprotective Effects of Red-Wine-Derived Compounds

In this section, we will recapitulate the main preclinical evidence found in the literature for the effects of red wine and its components on brain health. We will specifically explore the primary mechanisms through which polyphenols exert their effects, starting with oxidative stress.

As already highlighted, oxidative stress is a relevant mechanism in several neurodegenerative diseases associated with cognitive disorders. In this context, compounds derived from red wine have shown a beneficial effect in several preclinical studies.

Starting from in vitro studies, red wine constituents and, in particular, flavonoids showed neuroprotective properties against oxidative stress, improving cell viability by acting on DNA replication and repair, increasing intracellular glutathione, directly lowering levels of reactive oxygen species (ROS), modulating several oxidative-stress-sensitive pathways, such as Nrf2, and preventing cardiolipin oxidation, mitochondrial fragmentation and dysfunction, and the influx of Ca2 [83,84,85,86,87,88,89].

Some in vivo data also confirm the antioxidant properties of red-wine-derived polyphenols, particularly anthocyanins. These compounds enhance glutathione levels and modulate several pathways associated with oxidative stress, such as the already-mentioned Nrf2 pathway [90,91].

However, it is important to note that in the study conducted by Gian C. Tenore and colleagues [92], red wine polyphenols were found to be associated with decreased expression of transthyretin in the murine choroid plexus. Transthyretin is a well-known neuroprotective factor and a sensor of oxidative stress.

Additionally, numerous preclinical studies, both in vitro and in vivo, demonstrated the neuroprotective efficacy of resveratrol against oxidative stress. Resveratrol has been shown to reduce glutamate toxicity in acute hippocampal slices by modulating ROS production, preventing mitochondrial dysfunction, and regulating glutamine synthetase activity [93].

Related to in vitro studies, the beneficial effects of resveratrol on oxidative stress have been confirmed in several models, including an acute oxidative stress model with Caenorhabditis elegans, where it also influenced lifespan [94].

The antioxidant activity of resveratrol in vivo appears to be primarily associated with the activation of several antioxidant enzymes, such as sirtuine 1 (SIRT1), heme-oxygenase-1, and peroxiredoxin-2 [95,96,97]. It is also noteworthy that in the article by N. Khodaie and colleagues [97], the antioxidative action of resveratrol via peroxiredoxin-2 is synergistically enhanced when combined with moderate concentrations of ethanol.

Other compounds derived from red wine have also been shown to be effective in preclinical studies in preventing oxidative stress, such as quercetin, procyanidin B2, and ethyl ferulate, with mechanisms similar to those outlined above [98,99,100,101].

Another mechanism by which compounds derived from red wine act in the context of neurocognitive diseases in the preclinical setting is neuroinflammation. In this regard, the vast majority of studies are specific to resveratrol. Resveratrol has been shown to reduce lipopolysaccharide (LPS)-induced cortical neurotoxicity in in vitro studies, also acting on microglia activation by inhibiting their production of pro-inflammatory cytokines and matrix metalloprotease and by also inhibiting prostaglandin E2 and NO production [102,103,104,105].

The effects of resveratrol on neuroinflammation are also confirmed in mouse models by acting on formyl peptide receptors 1 and SIRT1 [106].

Regarding the polyphenol myricetin, in vitro data have shown that it can reduce microglia activation toward the M1 pro-inflammatory phenotype [107]. Specifically, it appears to exert this effect by inhibiting the signal transducer and activator of transcription 1 (STAT1) [107].

In addition to the mechanisms described above, compounds derived from red wine are found to have direct neuroprotective action in the preclinical setting. Several polyphenols have been demonstrated to mimic neurotrophins’ effects in vitro, together with a positive action on the neuronal cytoskeleton and synaptic plasticity, especially through phospholipase C and protein kinase C [108,109].

Among the various compounds in red wine, resveratrol is the most extensively studied in this area. In vitro data showed that resveratrol acts in a neuroprotective sense by reducing apoptosis pathways, as well as by reducing glutamate and cadmium toxicity [110,111,112].

In vivo studies have also demonstrated that resveratrol has a neuroprotective effect, especially against excitotoxic brain damage [113,114,115]. It also inhibits the postsynaptic glutamate receptors and increases BDNF at the serum level [113,114,115].

In addition, other compounds derived from red wine have also been shown to have similar direct neuroprotective actions both in vitro and in vivo, such as ellagic acid, gallic acid, quercetin, and tannic acid [116,117,118,119].

It is important to note that, consistent with the study by Tal Frolinger and colleagues [120], some of the effects mentioned above of grape-derived polyphenols in vivo are mediated by the gut microbiota, which influences their bioavailability.

However, some work shows that red wine may have a contribution to the production of potentially neurotoxic compounds, such as methanol or formaldehyde [121].

### 3.3. The Impact of Red Wine and Its Components on Mild Cognitive Impairment

The term MCI refers to a prodromal period that comes before the onset of dementia [1]. This heterogeneous clinical syndrome is characterized by alterations in specific cognitive domains among memory, attention, executive functions, language, and visuospatial skills [1].

Patients with amnestic MCI have a greater memory deficit than others of the same age but continue to function independently, though with less efficiency. There is growing evidence that even age-associated memory impairment, originally conceptualized as “benign forgetfulness,” can reflect very early neurodegeneration, especially for AD [122].

MCI carries a 50% risk of evolving into dementia within five years [1]. The early identification of MCI is crucial to implementing tailored therapeutic strategies in the effort to slow down the neurodegenerative process.

From this perspective, resting-state functional magnetic resonance imaging (fMRI) has proven to forecast cognitive or emotional behavior [123], identifying early alterations in brain activity associated with AD-related cognitive decline [37,124].

*Clinical evidence*—In 2009, Robert Krikorian and colleagues [125] conducted one of the first controlled human trials, demonstrating how the daily consumption of Concord grape juice (CGJ), an extract from grapes, could improve learning memory in older adults with early memory decline. These results were linked with greater fMRI activation in the anterior and posterior regions of the right hemisphere [33], a result linked to a greater hemodynamic response and increased neuronal activity due to CGJ supplementation [23,126,127]. Similarly, the administration of a polyphenol-rich grape and blueberry extract has been shown to improve the speed of information processing and visuospatial learning [128,129].

Successively, many other studies investigated the effects of dietary polyphenols on cognitive functions in MCI patients. Resveratrol was confirmed to be one of the main phenolic compounds able to have a significant effect on improving cognitive functions [1]. In Francesco Poti and colleagues’ study [1], the administration of resveratrol significantly enhanced verbal learning memory, visuospatial ability, and executive functions but failed to show a significant amelioration in global cognitive functions. Conversely, these results are in contrast with another meta-analysis conducted by Mohammad Hosein Farzaei and colleagues [26], where the beneficial effects of polyphenol administration were demonstrated on mood symptoms rather than on memory and cognitive performance. This discrepancy may be related to the different enrollment criteria and very high interindividual differences in resveratrol bioavailability.

In their evaluation study, Lucas Zoppi Campane and colleagues [130] investigated the long-term effects of red wine consumption on the brain using fMRI 3.0 Tesla magnetic resonance system (Philips Achieva). Red wine consumers showed greater activation within the posterolateral portions of the right thalamus and in the posterior portion of the ipsilateral insula, areas that participate in the processing of visual stimuli. The group consuming red wine also showed a negative correlation between average daily ethanol consumption and increased activation of these subcortical structures, suggesting that alcoholic beverages may subtly modify the activation of these brain regions.

Among abstainers, greater activation was observed in cortical areas in the left superior parietal lobule and homolateral angular gyrus, both involved in working memory and integrating information processes. On the other hand, there was no difference in periventricular and deep cerebral white matter compartments between the two groups, suggesting that regular red wine consumption may not induce beneficial encephalic vascular effects [130].

However, several epidemiological studies instead suggested that light-to-moderate consumption of red wine may protect against cognitive decline, as individuals who drink about 1.5 glasses of red wine per day are more likely to show a lesser decline in global cognitive function compared to those with lower red wine consumption [36].

### 3.4. The Impact of Red Wine and Its Components on Alzheimer’s Disease

AD is the most common form of dementia and may contribute to 60–80% of cases [70], with a greater proportion in the higher age ranges [9].

The familial early-onset form of AD is caused by mutations in the genes amyloid precursor protein (APP) and presenilin 1 and 2. The APOE gene is associated with an increased risk for the sporadic form of the disease. In the pathophysiology of AD, neuronal and synaptic loss begins in the hippocampus, the brain region that is involved in memory and learning, leading to its atrophy. As the pathology progresses, it spreads to other brain areas, such as the amygdala and basal forebrain, and then affects the entire brain [9].

AD is characterized by a specific neuropathology hallmark: extracellular senile plaques, composed of the accumulation of abnormally misfolded Aβ peptides, tangle intracellular neurofibrillary formation (for which hyperphosphorylated tau is the major protein), and amyloid angiopathy [131].

The pathophysiology of the disease is complex and probably involves multiple overlapping and redundant pathways of neuronal damage, which leads to selective neuron loss and shrinkage, synapse loss, and disruption of cholinergic neurotransmission [9]. Although the primary cause of AD remains unknown, several lines of evidence suggest the involvement of oxidative stress [132].

Aβ-induced changes are believed to occur in the earliest stages of AD, a long time before the impairment of cognitive functions appears. When symptoms occur, there has already been a substantial loss of neurons, and it is only possible to counteract the symptoms [1,9]. The initial symptoms of AD are usually progressive memory loss, cognitive function decline, and behavioral and psychological symptoms [9].

Asymptomatic carriers of AD genetic risk factors, such as the apolipoprotein E ε4 allele (APOE4) or a parental family history of AD, tend to show neural changes and cognitive decline in executive function over time [133]. The recognition of this subtle cognitive impairment may serve as an early cognitive marker of AD [134].

Below, we discuss current evidence from the literature on the effect of red wine and its compounds on this disease, starting with preclinical data.

*Preclinical evidence*—Starting from red wine per se, its moderate consumption significantly improves memory impairment together with AD neuropathology in the mouse model [135]. A possible explanation of its effect is associated with the promotion of the nonamyloidogenic processing of APP [135].

Data on Muscadine wine confirmed the positive effects reported above in a mouse model of AD, but with a possible different mechanism of action [136]. Specifically, it appears to reduce the accumulation of soluble, high-molecular-weight oligomeric Aβ species in the brain by interfering with the aggregation of Aβ peptides [136].

Regarding grape-derived polyphenols, in vitro studies showed inhibitory effects on Aβ assembly, protofibril formation, and its oligomerization [137,138,139,140]. Together with this, it was also able to reduce the overall cytotoxicity of Aβ by acting on apoptotic features, intracellular ROS accumulation, reducing DNA fragmentation and lipid peroxidation, and increasing the cellular glutathione pool [139,141]. The study by Belgin Sert Serdar and colleagues [142] showed that a combination of different polyphenols could pass through an in vitro model of the BBB and disaggregate Aβ. Notably, this combination of different polyphenols was more effective in achieving these effects than single polyphenols alone [142].

Relative to in vivo studies, grape-derived polyphenols were also able to improve the neuropathology of AD in mouse models, together with a reduction in neuroinflammation, probably by also acting on extracellular signal-receptor kinase 1/2 signaling in the brain [143,144,145,146].

The effect of polyphenols derived from grapes and red wine on Aβ appears to be related to their ability to bind and stabilize its early oligomeric species, thereby inhibiting the formation of larger aggregates [147,148]. The study of Ali Reza A. Ladiwala and colleagues [149] demonstrated that while the effects of polyphenols differ depending on whether they are glycosides or aglycones, both forms are associated with a reduction in Aβ toxicity.

In some studies, although no effects related to this treatment on AD neuropathology have been observed in a mouse model, there is nonetheless an improvement in cognitive function, probably due to its action against the negative cytotoxic effects associated with proteinopathy [150].

Finally, the study by Craig P. Hutton and colleagues [151] incorporated polyphenols found in grapes and red wine, along with other dietary components that affect AD. This approach allowed these aspects to be studied in a more translational context, where red wine is part of an overall diet. They found that short-period supplementation with this multi-ingredient dietary supplement was able to partially improve cognitive impairment in a mouse model of AD, in particular in working memory and spatial learning [151].

The most relevant and studied single red wine polyphenol in AD is resveratrol. Preclinical studies, both in vitro and in vivo, provide evidence of its benefits for AD.

Starting from in vitro studies and focusing on the resveratrol effect on Aβ, the most common effect described is the reduction in its cytotoxicity [152,153,154]. This neuroprotective effect is attributed to various mechanisms, such as reducing oxidative stress through the upregulation of HO-1, interfering with Aβ fibril formation and apoptotic pathways, inducing autophagy, addressing mitochondrial dysfunction, and reducing nuclear factor kappa-light-chain-enhancer of activated B cells (NF-κB) activation [152,153,154,155].

Focusing on the Aβ resveratrol direct effects, there are also, in this case, several described mechanisms. It is described, in fact, that resveratrol is able to disrupt Aβ1–42 aggregation by inducing its fragmentation into smaller peptides and, according to molecular dynamics simulation, interfering with the Aβ17–42 pentamer [156,157].

Moreover, resveratrol affects Aβ-induced microglial activation by reducing its proliferation and release of pro-inflammatory cytokines [158].

Resveratrol seems to act not only directly and indirectly on Aβ but also on tau. In detail, it is described as a resveratrol-mediated reduction in the hyperphosphorylation of tau by suppressing glycogen synthase kinase (GSK-3β) and calmodulin-dependent protein kinase II (CaMKII) activities and by increasing the activity of phosphoseryl/phosphothreonyl protein phosphatase-2A (PP2A) [159,160]. It is important to note that viniferin, a dimer of resveratrol, shows similar beneficial effects on AD in in vitro studies [161,162].

Discussing the in vivo data, the previously described in vitro effects of resveratrol are largely confirmed across several AD models. Overall, resveratrol enhances cognitive function and mitigates AD pathology [163,164,165,166].

Specifically, resveratrol has been shown in vivo to directly influence Aβ, reducing amyloid plaque formation through various mechanisms. Literature reports indicate that it modulates APP processing by also favoring the non-amyloidogenic pathway and activating autophagy and proteasomal degradation pathways, at least partially via AMP-activated protein kinase (AMPK) activation [167,168,169,170,171,172,173].

Moreover, resveratrol acts in vivo on the indirect effects of Aβ by reducing the brain inflammatory status, reducing oxidative stress by enhancing intracellular glutathione, and reversing Aβ1–42-induced decreases in the activity of cAMP response-element binding protein (CREB), BDNF, and anti-apoptotic factor BCl-2 expression [174,175,176,177].

Regarding tau, in the study of Francisco Alejandro Lagunas-Rangel, a bioinformatics analysis was performed, showing an inhibitory effect of resveratrol on the activity of cyclin-dependent kinase 5, with possible effects on tau phosphorylation [178]. At the same time, in vivo studies have shown that resveratrol affects its hyperphosphorylation via PP2A. This effect is attributed to a resveratrol-mediated reduction in the expression of the MID1 ubiquitin ligase, which plays a role in the degradation of the catalytic subunit of PP2A [179].

Another important element of red wine studied in AD is tannic acid. Several preclinical studies have shown its positive effect against this disease, both in vitro and in vivo. Starting from in vitro studies, it has been shown to be a potent inhibitor of Aβ42 and tau aggregation, as well as to modulate major pathways of ferroptosis, such as iron chelation, inhibition of lipid peroxidation, rescue of mitochondrial damage, and activation of the Nrf2 axis [180,181,182].

Furthermore, the in vivo study conducted by Mariana F. B. Gerzson and colleagues on a rat model of AD demonstrated how tannic acid improves cognitive status, especially in areas relative to learning, recent memory, and spatial recognition. It also reduces neuronal death, restores total and phosphorylated Akt levels, and reduces neuroinflammation [183].

Two other relevant polyphenols with actions on AD described in the literature are quercetin and epicatechin. Preclinical studies, both in vitro and in vivo, have shown that these polyphenols directly affect Aβ and tau pathology, as well as oxidative stress, microgliosis, and astrocytosis [184,185,186,187,188,189].

It is also interesting to note that in the study of Amin Molaei and colleagues [186], the positive quercetin effects on AD were further emphasized by the association with physical exercise, showing how an overall lifestyle modification can be an effective strategy toward this disease.

Other components of red wine, such as chlorogenic acid, ellagic acid, p-coumaric acid, phenolic acids, anthocyanins, fisetin, morin, myricetin, piceid, and rutin, have shown effects in several preclinical articles pertaining to AD [190,191,192,193,194,195,196,197,198,199]. However, the evidence is less robust due to the limited number of studies.

*Clinical evidence*—Regular consumption of flavonoid-rich food and drink has been linked to a 50% reduction in the risk of dementia, protection of cognitive performance with aging, and a delay in the development of AD [19,200,201,202,203,204].

It is noteworthy that the neuroprotective properties against the formation of these beta-amyloid plaques are performed by some grape polyphenols, such as resveratrol, catechin, and quercetin [13,205,206].

A light-to-moderate consumption of red wine in people with AD or dementia seems to be protective against cognitive decline [36,207,208,209,210]. This appears to be true, especially in men, as women can be more susceptible to the detrimental effects of alcohol [28]. However, it is well known that exceeding moderate red wine consumption potentially nullifies these benefits and leads to adverse health outcomes, reflecting a U-shaped dose–response relationship [28,36,211].

Many studies have investigated how APOE4 status and parental or family history of AD may modify how food is related to cognition over time.

People without a family history of AD are more likely to benefit from the neuroprotective effect of red wine consumption, as it has been demonstrated to enhance left executive function connectivity in the central executive function network upon fMRI. In contrast, individuals with a family history of AD displayed reduced network connectivity in relation to their red wine consumption [37].

In the literature, there are divergences regarding the link between APOE status, wine consumption, and the risk of dementia. Many studies suggest that APOE4 carriers with higher alcohol consumption are more likely to develop dementia, while in APOE4 non-carriers, daily wine consumption is associated with a lower risk of AD [210]. However, these results are in contrast with Brandon S. Klinedinst and colleagues’ [212] findings, where daily red wine consumption predicted higher fluid intelligence levels over time only for APOE4+ adults with AD family history. The influence of APOE4 status on diet needs to be further investigated.

### 3.5. The Impact of Red Wine and Its Components on Vascular Dementia

Evidence suggests that vascular factors not only contribute to age-related cognitive decline but also play a role in the two most prevalent dementias: AD and VaD. VaD represents 15–30% of overall dementia, as cognitive impairment and cardiovascular diseases are linked by their risk factors, which may include hypertension, hyperlipidemia, coronary artery disease, and stroke [10,213].

Aging-related vascular issues, such as strokes and small vessel disease, further underscore the connection between brain vascular circulation and cognitive function. The competence of the microvasculature to meet metabolic demands decreases with age, potentially impacting adult neurogenesis [4,5]. White matter lesions, or hyperintensities, are linked to an increased cardiovascular risk [213,214]. Reduced cerebral blood flow and vascular density are common indicators, though it is uncertain whether white matter lesions cause vessel loss, or vice versa.

Recent findings indicate a significant overlap between AD and VaD. The beta-induced damage at the endothelial level can alter the processes of repair and regeneration, in turn accelerating the progression of deposition in vascular dementia [215]. Furthermore, high blood pressure has been linked to increased neurofibrillary tangles, a hallmark of AD. Postmortem studies have shown that 77% of VaD cases exhibited AD pathology [216].

*Preclinical evidence*—According to our search, there are limited preclinical data in the literature regarding the effect of red wine and its components on cerebrovascular damage. First of all, in the study of Chen Chen and colleagues [217], a one-month treatment with grape seed polyphenol extract showed positive effects in a rat model of chronic cerebral hypoperfusion. In particular, this treatment was able to rescue memory impairment and cholinergic dysfunction without altering any main physiological parameters. Moreover, it reduced the oxidative stress caused by malonic dialdehyde, together with an increase in antioxidant enzymes at the hippocampus level.

Regarding resveratrol, it has a positive effect on both acute and chronic cerebrovascular damage. Relative to acute injury, Zhen Li and colleagues [218] showed that pretreatment with resveratrol reduced ischemia-induced cerebral infarction together with ischemia-impaired spatial memory in a rat model of ischemic stroke. This effect was mediated by the activation of the N-methyl-D-aspartate (NMDA) receptor and the CREB signaling pathway. Regarding chronic vascular damage, Veysel Haktan Ozacmak and colleagues [219] demonstrated the neuroprotective effect of resveratrol in a model of chronic cerebral hypoperfusion using ovariectomized female Wistar rats. This effect was evident in both the hippocampus and cortex, accompanied by a decrease in lipid peroxidation and a restoration of reduced glutathione levels. Other authors showed the protective effect of resveratrol in a rat model of chronic cerebral hypoperfusion by improving cognitive impairment, synaptic transmission, and spinogenesis, together with a reduction in apoptotic pathways [220,221]. These effects are at least partially mediated by the CREB signaling pathway [220].

In animal models, quercetin has also been shown to improve endothelial function and lower blood pressure through its angiotensin-converting enzyme inhibitory activity and by increasing NO bioavailability [222].

*Clinical evidence*—A polyphenol-rich diet has been associated with better cognitive function in older adult subjects at high cardiovascular risk [223]. Regular and moderate consumption of red wine has been correlated with a lower incidence of atherosclerosis among the French (the so-called “French paradox”) [224]. Flavanols have been demonstrated to increase NO synthesis, which can enhance overall vascular function, including brain circulation, lower blood pressure, improved endothelial function, and reduced LDL cholesterol oxidation [33].

We discussed earlier the benefits of quercetin in preclinical studies. However, human studies have been less convincing, possibly due to the lower bioavailability of quercetin in human tissue and cells [222].

### 3.6. The Impact of Red Wine and Its Components on Parkinson’s Disease Dementia

Parkinson’s disease (PD) is a common movement disorder characterized by a significant and targeted depletion of dopaminergic neurons within the substantia nigra pars compacta [30]. This depletion is reflected in motor dysfunction, with symptoms such as bradykinesia, rigidity, resting tremor, postural instability, and gait disturbances [30]. The hallmark of pathology in PD is Lewy bodies, which are intracellular inclusions made by the accumulation of the presynaptic soluble protein α-synuclein [30].

PDD refers to a decline in cognitive function and other mental abilities that often accompanies PD. It is estimated that up to 83% of people with Parkinson’s disease will eventually develop dementia in 20 years as their condition progresses. Together with the spread of α-synuclein, two key processes that may contribute to cognitive decline in PD are tau accumulation and neuroinflammation [225]. Symptoms of PDD can include memory loss, confusion, difficulty with problem-solving, hallucinations, and changes in mood and behavior [225].

*Preclinical evidence*—Also in this context, there are studies in the literature demonstrating the effects of grape polyphenols and polyphenol-rich extracts in both in vitro and in vivo models of PD [226,227,228]. In in vitro models, anthocyanins and other polyphenol-rich extracts exhibited neuroprotective effects primarily due to the activity of the antioxidant transcription factor Nrf2 and their action on α-synuclein fibrillation [226,228].

In in vivo models, grape polyphenol concentrates reduced α-synuclein accumulation, decreased neuroinflammation, extended lifespan, and improved PD symptoms [222,227].

Regarding resveratrol, it has also shown a neuroprotective effect in preclinical models of PD. This was described in an in vitro model, where resveratrol reduced neuronal cell death by modulating apoptotic proteins, microglia pro-inflammatory activity, and the oxidative state [229,230].

In the in vivo models, resveratrol was able to improve both the motor and cognitive status by inhibiting α-synuclein aggregation and cytotoxicity, increasing the level of tyrosine hydroxylase, attenuating neuroinflammation, and improving the oxidative status through its free radical scavenging capacity [231,232].

Other red-wine-associated polyphenols, such as ellagic acid and quercetin, showed neuroprotective effects on α-synucleinopathies in in vitro models, also in this case by reducing α-synuclein aggregation and modulating apoptotic proteins, but also acting on autophagic clearance [229,233,234,235]. Focusing on quercetin, Fatemeh Ghaffari and colleagues [236] showed that this molecule had positive effects on cognitive impairment and reduced oxidative stress in the hippocampal area in a PD mouse model [236].

*Clinical evidence*—Few human studies are present in the literature on the relationship between PDD and red wine. Additionally, there are even fewer preclinical and clinical data for other significant α-synucleinopathies associated with cognitive decline, such as DLB.

### 3.7. The Impact of Red Wine and Its Components on Metabolic Syndrome and Diabetic-Associated Cognitive Impairment

The relationship between impaired executive functions and obesity and, more generally, metabolic syndrome has been documented in the literature [237]. Obesity is characterized by excessive adiposity and elevated levels of pro-inflammatory adipokines, such as tumor necrosis factor alpha and interleukin-6, resulting in a chronic low-grade inflammatory state. These biochemical alterations contribute to neuroinflammation, neuronal damage, gliosis (fibrosis of brain tissue), and neuronal cell death, potentially leading to cognitive impairment [237].

*Preclinical evidence*—Also in this context, preclinical data show that compounds derived from red wine and grapes could have beneficial consequences. Grape polyphenol preparations have had positive effects on mouse models of metabolic syndrome, not only with respect to parameters such as glucose tolerance and blood pressure but also on synaptic impairments at the hippocampal level [238].

We also know that type II diabetes mellitus is another relevant risk factor for developing neurocognitive disorders, potentially having direct negative effects on the brain.

Beneficial effects have also been shown for resveratrol in this context. Specifically, in vitro data show how resveratrol is able to protect neuronal cells exposed to high glucose by activating PI3K/Akt/FoxO3a, reducing oxidative stress, and preventing the development of apoptosis [239]. In a similar model, also in vitro, quercetin was also able to reduce oxidative stress and apoptotic processes [240,241].

In vivo data confirm the benefits of resveratrol at this level, acting in animal models at the brain level on oxidative and inflammatory status, reducing synapse loss, and improving the overall cognitive picture [242,243].

*Clinical evidence*—In clinical studies on obese or overweight patients without cognitive decline, the neuroprotective effect of resveratrol has been documented only in young and middle-aged adults [37,244], while in older adult participants, there were no significant effects [245,246].

Sebastian Huhn and colleagues studied the effect of a daily administration of resveratrol (200 mg) plus quercetin (320 mg) on cognitive status. The study failed to demonstrate a significant improvement in cognitive performance, but while the placebo group showed a decline in spatial working memory, the resveratrol group did not [245]. These results may suggest that resveratrol helps preserve cognitive function, while memory gradually declines with aging [247]. In contrast, Hamish M. Evans and colleagues enrolled a younger population than Sebastian Huhn and colleagues and demonstrated how the administration of 150 mg/day of resveratrol to postmenopausal women had a significant effect on improving executive function, attention, and cognitive processing speed [244].

The role of age in mediating the effects of polyphenols in obese or overweight people at risk of cognitive impairment is something that needs further investigation [237].

There are also discrepancies in the literature regarding the effects in vivo of resveratrol administration on glucoregulation and its link with cognitive performance.

Anja Veronica Witte and colleagues demonstrated how the administration of resveratrol was able to reduce glycated hemoglobin A1c (a marker of long-term glucose levels), with an improvement in episodic memory and in hippocampal and medial prefrontal cortex connectivity [248]. However, these results are in contrast with Sebastian Huhn and colleagues’ study, where HbA1c levels, as well as the hippocampus volume, microstructure, and functional connectivity, did not change significantly, compared to the placebo group, after the administration of resveratrol [245].

### 3.8. Preclinical Data on the Effect of Red Wine Compounds on Other Neurocognitive Disorders

In this final section of the results, we discuss preclinical evidence regarding the effects of red wine components on other types of neurocognitive disorders, beginning with age-related cognitive decline.

Starting with grape extracts, the data in this area are predominantly from in vivo studies in animal models of aging. These interventions have shown effects on cognitive function, in particular spatial learning and memory, by influencing mitochondrial activity, hippocampal nerve growth factor, neuroinflammation, proteostasis, and oxidative stress, partly by increasing SOD levels [249,250,251,252,253,254]. These beneficial effects are also observed in animals that have consumed a high-carbohydrate, high-fat diet [254].

Among the components of red wine, certainly most studied in this context is resveratrol. Research from animal models of aging indicates that resveratrol has a positive effect on locomotor activity and short- and long-term memory. This effect seems mainly due to the reduction in oxidative stress and, consequently, lipid peroxidation, as well as the inflammatory state. These effects of resveratrol are probably primarily mediated by its action on adenosine-mediated signaling, CREB, and neurotransmitter homeostasis [255,256,257,258,259,260].

Another area of interest where polyphenols in red wine have been shown to have positive effects is cognitive impairment associated with sleep deprivation. These data come mainly from preclinical, in vivo data. In particular, grape polyphenols have been shown to alleviate cognitive deficits by activating CREB and mechanistic target of rapamycin (mTOR) pathways, promoting synaptic plasticity [261,262].

Relative to chronic stress-induced cognitive impairment, resveratrol led to an overall cognitive enhancement in this context through its action on SIRT1 and the BDNF/CREB pathway [263,264].

Finally, resveratrol has been shown in vitro to have neuroprotective effects in different chemotherapy-induced cognitive impairments by acting on neuroinflammation and BDNF levels, especially hippocampal levels [265,266].

**Figure 2:**
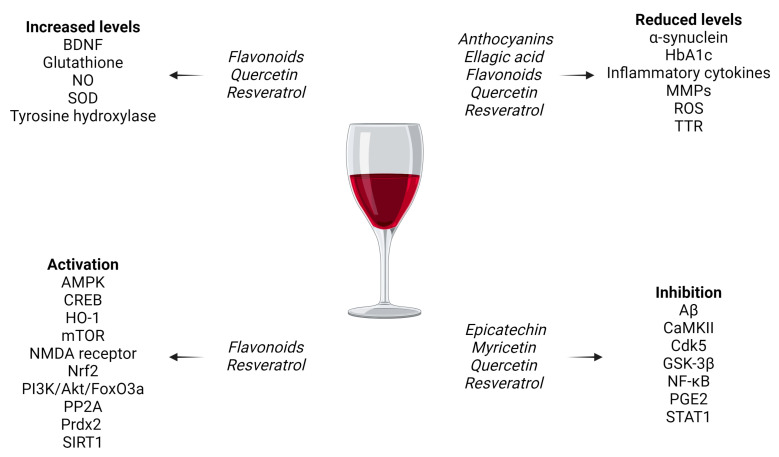
Main molecules targeted by red wine components in preclinical studies. Abbreviations: BDNF (brain-derived neurotrophic factor), NO (nitric oxide), SOD (superoxide dismutase), AMPK (AMP-activated protein kinase), CREB (cAMP response-element binding protein), HO-1 (heme-oxygenase 1), mTOR (mechanistic target of rapamycin), NMDA (N-methyl-D-aspartate), PI3K (phosphoinositide 3-kinases), FoxO3a (forkhead box O3), PP2A (protein phosphatase 2), Prdx2 (peroxiredoxin 2), SIRT1 (sirtuine 1), HbA1c (glycated hemoglobin), MMPs (matrix metalloproteases), ROS (reactive oxygen species), TTR (transthyretin), Aβ (amyloid β), CaMKII (calmodulin-dependent protein kinase II), Cdk5 (cyclin-dependent kinase 5), GSK-3β (glycogen synthase kinase 3β), NF-κB (nuclear factor kappa-light-chain-enhancer of activated B cells), prostaglandin E2 (PGE2), and signal transducer and activator of transcription 1 (STAT1). Created with BioRender.com.

See Figure 2 for the main effects of red wine component molecules associated with neurocognitive disorders and Table 2 for a synopsis of their effects specific to each disease.

### 3.9. Dose–Response Correlation between Alcoholic Beverages and Dementia

In conclusion, we address the issue of data in the literature regarding the dose–response effect of alcoholic beverages on dementia risk. Several meta-analyses show a nonlinear association between alcohol consumption and cognitive dysfunction and dementia, with light-to-moderate alcohol intake [267,268,269]. Specifically, the alcohol intake associated with a lower risk of dementia has been limited to a maximum of 12.5 g/day, with the risk bottoming out at about 6 g/day, while the risk increases when the intake exceeds [267]. This seems particularly relevant in adults younger than 60 years of age and more consistent with wine intake [267]. Furthermore, it appears that this association is more pronounced in men with AD, showing sex-specific effects [268]. The above association also seems to be supported in relation to the progression from MCI to dementia [270].

## 4. Discussion

The potential of red wine in the context of cognitive decline and dementia is a subject of considerable interest and debate within the scientific community. Red wine is rich in polyphenols, such as resveratrol, which possess antioxidant and anti-inflammatory properties that could theoretically protect against the cognitive decline associated with dementia [41,43]. Polyphenols, including resveratrol, have been shown in our review to neutralize free radicals, thereby reducing oxidative stress, which is a significant factor in the pathogenesis of neurodegenerative diseases, such as AD [139,141]. Oxidative stress leads to the damage of cellular components, including lipids, proteins, and DNA, which can impair neuronal function and survival. Furthermore, the anti-inflammatory properties of these compounds can mitigate neuroinflammation, another critical factor in the development and progression of dementia [106]. Chronic inflammation in the brain is associated with the activation of microglia, the brain’s resident immune cells, which can exacerbate neuronal damage and contribute to cognitive decline. The papers identified in our review have mostly demonstrated that resveratrol can inhibit the activation of these microglia, reducing the inflammatory response and potentially preserving cognitive function [158].

Moreover, light-to-moderate consumption of red wine has been associated with improved cardiovascular health, which is closely linked to brain health [271]. Some studies in our review have shown that moderate red wine intake can lead to better endothelial function and increased production of NO, a vasodilator that improves blood flow [3,17,18]. Enhanced cerebral blood flow ensures that the brain receives adequate oxygen and nutrients, which are essential for maintaining cognitive function and preventing neurodegeneration. Cardiovascular health is crucial in the context of vascular dementia, where impaired blood flow leads to cognitive decline.

Some other identified studies have provided evidence suggesting that resveratrol can activate certain signaling pathways that promote neuronal survival and plasticity. For instance, resveratrol has been found to activate the SIRT1 pathway, which is involved in cellular stress resistance and longevity [95,96,97]. Activation of SIRT1 has been shown to protect against neuronal damage and enhance cognitive function in animal models. Additionally, resveratrol has been reported to cross the BBB, directly exerting its protective effects on brain tissue [142]. Furthermore, the social and mental health benefits associated with moderate red wine consumption, such as relaxation and stress reduction, could also indirectly support cognitive health. Chronic stress is a known risk factor for cognitive decline and dementia, as it can lead to the release of glucocorticoids that negatively impact brain structures involved in memory and learning, such as the hippocampus [272].

Overall, the polyphenols found in red wine, based primarily on preclinical data, appear to exhibit not only neuroprotective properties but also a broader, pleiotropic effect on various aging-related processes linked to the pathophysiology of major neurocognitive disorders [273]. These include a reduction in oxidative stress and inflammation, and improvements in proteostasis and mitochondrial function, making them of great interest in the broader context of anti-aging effects. These effects on aging processes and longevity seem particularly relevant when there is a combination of multiple polyphenols [274], as is the case in red wine.

However, these potential benefits must be weighed against significant risks. Regular consumption of red wine can lead to alcohol dependency and abuse, which are associated with numerous health problems, including liver disease, cardiovascular issues, and an increased risk of accidents and injuries [275]. Excessive alcohol intake is neurotoxic and can cause brain damage and cognitive impairments [276]. The threshold between moderate and harmful consumption can be challenging to manage, particularly in older adults who may have a lower tolerance for alcohol. In 2020, 4.1% of all new cancer cases worldwide were attributable to alcohol consumption, and alcohol cessation is considered a relevant strategy in terms of prevention of these diseases [277,278].

Considering all these data, the World Health Organization stated that “no safe amount of alcohol consumption for cancers and health can be established” [279].

Collectively, red wine’s protective effects against dementia are inconsistent, with some studies showing benefits and others finding no significant impact or even potential harm. These mixed results, together with the expressed risks, highlight the need for more robust, long-term, and controlled studies to better understand the relationship between red wine and dementia risk. These complex and not entirely conclusive findings on light-to-moderate consumption of alcoholic beverages in general related to this matter were also highlighted in the 2024 update of the Lancet Commission on dementia [280].

Individual variability due to genetic, metabolic, and lifestyle differences further complicates the ability to make general recommendations. Factors such as genetic predisposition to alcohol metabolism, overall diet, and the presence of other health conditions can influence the effects of red wine on an individual basis.

Red wine is often included in the Mediterranean diet and may contribute to its potential benefits [281], which have been shown to significantly reduce the risk of dementia [11,12].

Furthermore, both red wine and the Mediterranean diet are commonly associated with social and convivial settings in the literature [282], which may play a role in lowering dementia risk, as social isolation is a known risk factor for the condition [280].

Polyphenols are easily available from other sources, such as fruits, vegetables, nuts, and teas, providing these beneficial compounds without the associated risks of alcohol.

As highlighted in our literature review—especially in preclinical settings—these molecules have shown the most compelling evidence for neuroprotective effects. Moreover, an increasing number of studies are beginning to demonstrate the potential positive impact of chronic supplementation of these compounds, particularly certain classes, such as flavonoids, on cognitive function [237]. However, it is important to note that currently, these data remain inconclusive.

In conclusion, when comparing our findings with other literature reviews, many of which lack a comprehensive literature search strategy, we similarly observed varied data on the potential neuroprotective effects of light-to-moderate red wine consumption and its components, particularly in conditions such as AD and vascular dementia [283,284]. However, several uncertainties remain, including the known risks associated with alcohol, individual predisposition, and the confounding effects of other lifestyle factors, as previously highlighted. Additionally, not fully resolved issues persist regarding the bioavailability of red wine polyphenols and the significance of the synergistic interactions between its various components [285,286].

The strengths of this article are the consideration of the effect of red wine and its components on a broad spectrum of neurocognitive disorders, as well as the evaluation of both preclinical and clinical data. In our opinion, this ensures a more comprehensive view of the topic.

The main limitation is certainly the lack of a fully systematic approach, given the breadth of aspects considered.

## 5. Conclusions and Future Perspectives

While red wine may offer some cognitive benefits due to its polyphenolic compounds, such as resveratrol, the potential risks, especially related to alcohol consumption, require careful consideration. The current body of evidence is not sufficiently robust or consistent to make definitive conclusions about its role in dementia prevention and treatment because it is mainly related to preclinical studies.

There is still scant evidence in humans of the effects of red wine consumption on cognitive decline in long-term studies, for which the results remain controversial. A possible explanation for the discrepancies in results may be found in the differences in study populations, dietary habits, and the diverse absorption rates of polyphenols, which are intricately influenced by factors such as the microbiota composition.

Therefore, there is currently insufficient evidence to indicate red wine intake with a view to dementia prevention, even at light-to-moderate doses. It is essential to approach red wine consumption with caution and to consider alternative sources of polyphenols and antioxidants that do not carry the risks associated with alcohol.

To advance our understanding, more methodologically robust longitudinal studies are needed, encompassing risk protection for neurocognitive disorders in diverse populations, accounting for potential confounding factors, such as social influences and dietary patterns, and considering general health risks linked to red wine consumption. Additionally, it will be crucial to personalize the assessment of risks and benefits for individuals regarding red wine intake, moving toward increasingly tailored health recommendations. Lastly, the exploration of other polyphenol-rich foods that lack the risks of alcohol, along with nutraceutical trials investigating specific compounds, such as resveratrol, which is already supported by emerging evidence in the current literature [287], will become of growing interest.

In conclusion, our literature review did not find conclusive evidence supporting the potential beneficial effects of chronic light-to-moderate red wine consumption on cognitive decline or dementia. However, there are promising data on the neuroprotective activity of polyphenols derived from red wine, though further research is needed to validate these findings.

## Figures and Tables

**Table 1:** Polyphenol content in red wine according to their classes and sub-classes [82].

Red Wine Polyphenols	Total Content (mg/100 mL)
*Flavonoids*	82.50
Anthocyanins	22.34
Dihydroflavonols	5.44
Flavanols	53.86
Flavanones	0.86
*Phenolic acids*	17.15
Hydroxybenzoic acids	7.00
Hydroxycinnamic acids	9.99
Hydroxyphenylacetic acids	0.16
*Stilbenes*	4.36
*Other polyphenols*	4.37
Hydroxybenzaldehydes	0.71
Tyrosols	3.66
*All polyphenols*	108.38

**Table 2:** The table describes the effects documented in preclinical studies of red wine polyphenols at the level of different molecular processes involved in neurocognitive disorders. Abbreviations: Aβ (amyloid beta) and NO (nitric oxide).

Polyphenol	Neurocognitive Disorders	Study Model	Study Period	Dosage	Molecular Effects
*Resveratrol*	Alzheimer’s disease	In vitro (SH-SY5Y)	48 h	1 μM	Effects on Aβ: disruption of Aβ1–42 aggregation by inducing its fragmentation into smaller peptides and interfering with the Aβ17–42 pentamer [156,157,167,168,169,170,171,172,173]
		In vivo (mice models)	2–10 months	0.35–1% and 1 g/kg (diet or water)	
		In vitro (N2a and SH-SY5Y)	30 min and 8 h	10–25 μM	Effects on tau: reduced phosphorylation and increased activity of phosphatases [159,160,178,179]
		In vivo (wild-type mice)	2 weeks	25 mg/kg (daily intraperitoneal injections)	
		In vitro (PC12 cells)	2–24 h	20–40 μM	Neuroprotective effects: a reduction in oxidative stress, microglial activation, apoptotic pathways activation, inhibition of neurotrophic pathways and mitochondrial dysfunction, and induction of autophagy [152,153,154,158,163,164,165,166,174,175,176,177]
		In vivo (mice models)	2–10 months	0.35–1% and 1 g/kg (diet or water)	
	Vascular dementia	In vivo (rat models)	1 h to 4 weeks	30–40 mg/kg (intraperitoneal injections) and daily oral dose of 25 mg/kg	A reduction in lipid peroxidation and apoptotic pathways’ activation, restoration of reduced glutathione levels, and improvement in synaptic transmission and spinogenesis [218,219,220,221]
	Parkinson’s disease dementia	In vitro (PC12 cells, SH-SY5Y, and dopaminergic neurons)	30 min to 72 h	0.1–60 μM	Inhibition of α-synuclein aggregation and cytotoxicity, a reduction in oxidative status and apoptotic pathways’ activation, augmentation of the level of tyrosine hydroxylase, and attenuation of inflammation [229,230,231,232]
		In vivo (rat and mice models)	1–5 weeks	20 mg/kg intravenous and 10–50 mg/kg/day	
*Quercetin*	Alzheimer’s disease	In vitro (primary neuron cultures)		0.1–5 μM	Direct effects Aβ and tau pathology and a reduction in oxidative stress, microgliosis, and astrocytosis [184,185,186,189]
		In vivo (rat models)	1 week	80 mg/kg (intraperitoneal injections)	
	Vascular dementia	In vitro (rat vascular smooth muscle)	20–90 min	1–100 μM	Improvement in endothelial function and a reduction in blood pressure through inhibition of angiotensin-converting enzyme activity and by increasing NO bioavailability [222]
		In vivo (rat models)	5–45 min	14.7 µmol/kg intravenous and 88.7 µmol/kg oral administration	
	Parkinson’s disease dementia	In vitro (PC12 cells)	3 h	0.1 μM	A reduction in α-synuclein aggregation, oxidative stress, and apoptotic pathways’ activation and incrementation of autophagic clearance [229,234,235,236]
		In vivo (rat models)	30–120 min to 4 weeks	25–100 mg/kg oral administration	
*Ellagic acid*	Alzheimer’s disease	In vitro (PC12 cells)	24 h	0.1–1 μM	A reduction in oxidative stress and apoptotic pathways’ activation and promotion of neurite outgrowth [191]
	Parkinson’s disease dementia	In vitro (SH-SY5Y)	1–48 h	25–150 μM	Reducing α-synuclein aggregation and apoptotic pathways, and incrementation of autophagic clearance [233]

## Data Availability

No new data were created or analyzed in this study. Data sharing is not applicable to this article.
